# Risk factors for rifampicin resistance tuberculosis among patients attending Directly Observed Treatments Centres in Southwestern Nigeria

**DOI:** 10.11604/pamj.2024.49.87.43958

**Published:** 2024-11-22

**Authors:** Simeon Cadmus, Victor Oluwatoyin Akinseye, Temitayo Olagunju, Angela Makolo, Olutayo Falodun, Oluwole Lawal, John Babalola, Eniola Cadmus, Othman Yasir, Muse Fadeyi, Bolaji Ahmed, Alberto Piubello, Nimer Ortuno Guiterrez, Osman El-Tayeb

**Affiliations:** 1 Department of Veterinary Public Health and Preventive Medicine, University of Ibadan, Ibadan, Nigeria,; 2 Damien Foundation Genomics and Mycobacteria Research and Training Centre, University of Ibadan, Ibadan, Nigeria,; 3 Nigerian Institute of Medical Research, Yaba, Lagos, Nigeria,; 4 Department of Chemical Sciences, Augustine University, Ilara-Epe, Lagos, Nigeria,; 5 Department of Computer Science, University of Ibadan, Ibadan, Nigeria,; 6 Department of Microbiology, University of Ibadan, Ibadan, Nigeria,; 7 Oyo State Ministry of Health, Oyo State, Ibadan, Nigeria,; 8 Department of Community Medicine, College of Medicine, University of Ibadan, Ibadan, Nigeria,; 9 Department of Medicine, Leeds Teaching Hospitals NHS Trust, Leeds, United Kingdom,; 10 Damien Foundation Belgium (DFB), Brussels, Belgium

**Keywords:** Rifampicin, drug resistance TB, tuberculosis, presumptive TB, risk factors

## Abstract

**Introduction:**

tuberculosis (TB) remains a disease of global health importance. GeneXpert has emerged as a useful tool for the diagnosis of drug resistant TB (DR-TB). We determined the risk factors associated with DR-TB among presumptive pulmonary TB patients.

**Methods:**

a cross-sectional study was conducted among presumptive TB patients attending Directly Observed Treatments (DOTs) centres in Southwestern Nigeria. Sputum samples were obtained from individuals with suspected pulmonary TB, subjected to GeneXpert as the first-line test and then culture. Data were analysed using STATA 12.

**Results:**

sputum samples were collected from 2,169 consecutive patients and processed. A greater proportion of the participants (52.14%) were female, most within the age range of 20-39 (38.36%) and 40-59 (36.93%) years. About two-thirds, 66.34% (1439/2169) were GeneXpert positive and of this, 47 (3.27%) were DR-TB. Overall, 44.04% (855/2169) samples were culture positive. 7.56% of the patients were HIV positive, while 19.50%, 1.52% and 61.96% were new, relapse and previously treated cases, respectively. Multivariate analysis identified case definition (OR=2.38; 95%CI: 1.92-3.03) and (OR= 8.33; 95%CI: 5.26-12.50) and HIV (OR= 1.85; 95%CI: 1.29-2.65) and (OR= 3.61; 95%CI: 2.59-5.02) based on GeneXpert and culture as important risk factors for TB and DR-TB infection among participants.

**Conclusion:**

we found a moderate level prevalence of DR-TB with gender, previous TB treatments, and HIV status as major factors associated with DR-TB among study participants.

## Introduction

The challenges posed by tuberculosis (TB) globally cannot be overemphasized. According to the 2022 World Health Organization (WHO) report, there was an estimated 10.6 (9.8-11.3) million morbidity in 2021, 1.4 (11.3-1.5) million deaths among HIV-negative individuals, and an additional 187,000 (158 K-218 K) deaths among HIV-positive people globally [1]. The Africa region alone accounted for 25% of the global incidence and Nigeria (accounting for 4.6%) was listed among eight countries with about two-thirds of the global burden. Although there has been a reduction in TB incidence worldwide, especially in the last six years, except in 2021, when the COVID-19 epidemic interrupted case notification and treatment interventions in 2020; most of the high TB burden countries, however, were not able to meet the 2020 milestone of the End-TB Strategy [1].

According to the World Health Organisation (WHO), the problem of TB is exacerbated by the emergence of drug resistance (DR) and multi-drug resistance (MDR)-TB which have constituted a major threat to TB control and a cause for public health concern. In 2022, out of 3.7 million bacteriologically confirmed pulmonary TB cases tested for rifampicin resistance (RR), 141,953 MDR/RR-TB and 25,038 pre-XDR-TB or XDR-TB were identified, giving a total of 166,991 cases [1]. Incidentally, there were an estimated 18,200 deaths due to MDR/RR-TB, pre-XDR-TB and XDR-TB [1].

Timely and accurate detection of infection is imperative for prompt patient management and efficient treatment outcomes. In developing countries, misdiagnosis and false-negative results are major problems frustrating the efforts channeled towards the control of TB. The reason for this gap has been adduced to limitations in diagnostic regimens available in low-and middle-income countries (LMIC) of the world [2]. The Ziehl-Neelsen (ZN) smear microscopy is the most commonly used diagnostic tool in LMIC. This has poor sensitivity, as it cannot detect up to 54.8% of pulmonary TB patients [3], and this sensitivity is further reduced in individuals with extra-pulmonary TB [4]. Further, multiple visits to the health facilities are required, thus increasing the likelihood of error in results generated, as well as its inability to differentiate drug-susceptible from drug-resistant strains of mycobacteria [5]. Although *Mycobacterium* culture is considered as the gold standard, there are the associated challenges of a longer period of results turn-around (2-6 weeks), proper infrastructure, technical expertise, and contamination [2,6].

Within the last few decades, some important and efficient diagnostic techniques have been developed that utilise nucleic acid amplification (NAAT). One of these methods is the GeneXpert MTB/RIF (Cepheid), a revolutionary tool that has been used to provide immediate point-of-care (POC) tests for TB patients in well-equipped settings. Although, the sensitivity of GeneXpert is 92%, and specificity 99%, its implementation in resource-poor settings has been hampered by costs, the need for an uninterrupted power source and specialized laboratory equipment [7,8]. The GeneXpert MTB/RIF (Xpert) assay has been endorsed by the WHO as a rapid molecular test for the diagnosis of pulmonary TB in LMIC [9,10]. The assay has been approved for the diagnosis of TB in respiratory specimens (particularly sputum samples). In adults and children with signs and symptoms of extrapulmonary TB, Xpert MTB/RIF and Xpert Ultra may be used in lymph node aspirate, lymph node biopsy, pleural fluid, peritoneal fluid, pericardial fluid, synovial fluid or urine specimens as the initial diagnostic test rather than smear microscopy/culture. In adults and children with signs and symptoms of TB meningitis, Xpert MTB/RIF or Xpert Ultra should be used in cerebrospinal fluid (CSF) as an initial diagnostic test for TB meningitis rather than smear microscopy/culture.

In countries with high TB burden, continuous and regular surveillance and monitoring of drug resistance (DR) through constant testing is important to evaluate the exact burden of DR-TB, especially among endemic populations. Nigeria, like most LMICs, is however faced with the challenges of prompt, rapid, and efficient diagnosis of TB especially in rural and hard-to-reach locations. Although not widely distributed, the introduction of GeneXpert (507 currently available nationwide based on the database of the National TB and Leprosy Control Programme) into the diagnostic regimen at various Directly Observed Treatment Short-course (DOTS) centres in Nigeria has greatly improved the quality of diagnosis of TB and provided more insight into the proportion of the burden that is DR. The southwestern region of Nigeria is one of the regions with the highest number of healthcare facilities, most of which are located in the urban areas. Apart from the fact that most of these facilities are not well equipped, there is grossly inadequate manpower to cater for the healthcare needs of the teaming population. The provision of modern/functioning diagnostic tools for endemic diseases like DR-TB will significantly improve timely case detection and disease management in this region and by extension other regions of the country. Early detection and treatment of DR-TB is an important step towards the eventual control of the disease, most especially in high burdened regions of the world.

This study therefore investigated the prevalence and risk factors associated with DR-TB among presumptive pulmonary TB patients attending DOTs centres in Southwestern Nigeria. More specifically, this study aimed to explore the socio-demographic and clinical characteristics of TB patients, determine the prevalence of TB and DR-TB, as well as identify the risk factors associated with DR-TB among presumptive pulmonary TB patients attending DOTs centres in Southwestern Nigeria.

## Methods

**Study design:** this is a cross-sectional study, carried out among presumptive TB patients attending DOTs centres Oyo State, Nigeria.

**Figure 1:**
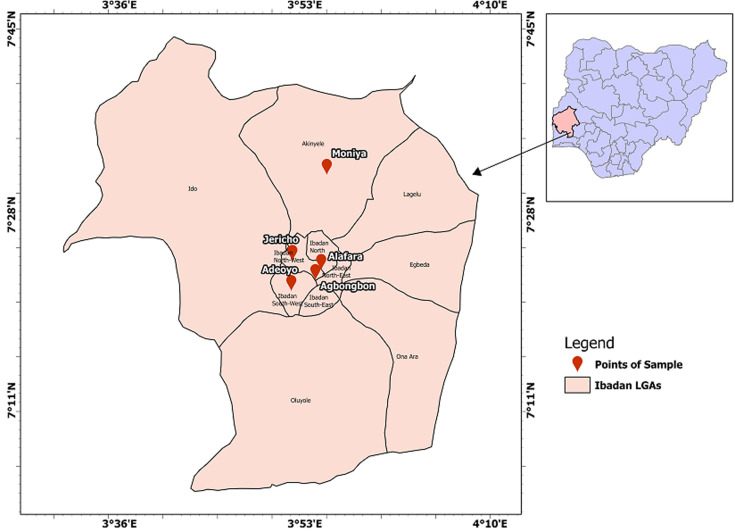
map of Ibadan Metropolis showing the five points of sample collection (inset Oyo State and Nigeria)

**Study site/setting:** the study was carried out, between October 2020 and May 2022, in selected DOTS centres in Ibadan, Oyo State (Figure 1), Southwestern Nigeria. The five health facilities selected for this study are distributed within the city. These DOTs centres are the major health facilities that handle TB cases in Oyo State (with an estimated population of about 6 million) [11]. Thus, they serve as the hub of TB care in the city of Ibadan and witness an appreciable level of patient visits daily.

**Study participants/sampling procedure:** all consenting presumptive/suspected pulmonary TB patients visiting the selected health facilities during the period were included in the study. However, those who were on treatment of any kind at the commencement of the study, and patients incapable of producing sputum were excluded from the study. Each participant was told to void sputum into a well-labeled capped cup in duplicate. The sputum sample was collected with a clean 50 ml capacity, sterile, disposable, polypropylene centrifuge, translucent, screw-capped container. Further, relevant epidemiological information obtained using a pre-designed form. At the point of collection, sputum samples were subjected to microscopy and GeneXpert to determine the TB infection status of the patients. The remaining sputum (second part) collected were transported daily in a cold chain to the Damien Foundation Genomics and Mycobacteria Research and Training Center (DF-GMRTC), University of Ibadan and either processed immediately for culture or stored at 4°C, prior to processing.

**Informed consent:** verbal consent was obtained from each intending participant, after careful explanation of the study protocol. Those who agreed to participate in the study were recruited and given a sputum cup to produce their sputum, while those who disagreed were excluded from the study.

### Operational definitions

**New TB patients:** patients that have never been treated for TB or have taken anti-TB drugs for less than one month [12].

**Relapse/recurrent patients:** individuals that have previously been treated for TB, and were declared cured or treatment completed at the end of their most recent course or treatment, and are now diagnosed with a recurrent infection, either as a result of true relapse or the new episode of TB reinfection [12].

**Previously treated patients:** these are a set of individuals who have received one month or more anti-TB drugs in the past and whose treatment failed or are declared loss to follow-up at the end of their most recent course of treatment [12].

**Pre-XDR-TB:** resistant to rifampicin and any fluoroquinolone, a class of second-line anti-TB drug.

**XDR-TB:** resistant to rifampicin, plus any fluoroquinolone, plus at least one of the group A drugs (i.e. bedaquiline and/or linezolid).

**Variables:** the independent variables/covariates in this study include gender, age group, case definition and HIV status; while the dependent/outcome measures are culture and drug susceptibility condition (measured by GeneXpert assay).

**Laboratory analyses:** sputum from presumptive pulmonary TB patients was checked for AFB using the Ziehl-Neelsen method in the health facilities and Xpert MTB/RIF assay (Cepheid, Sunnyvale, CA, USA) was also carried out. One of the duplicate samples was used for AFB microscopy and GeneXpert assay while the other was processed for culture. In addition, the X-ray information of individuals submitting samples was obtained. For each of the laboratory procedures carried out in this study, quality controls were conducted.

**GeneXpert assay:** the GeneXpert MTB/RIF (Xpert) is a rapid molecular test for the diagnosis of pulmonary TB in LMIC, and has been endorsed by the WHO. This assay allows for the simultaneous detection of *M. tuberculosis* complex and the most common mutations in the *rpoB*gene which confers resistance to rifampicin in less than 2 hours [9,10]. It is a nucleic acid amplification method that has been designed as an integrated DNA extraction and real-time polymerase chain reaction (PCR) system that is safe and simple to use. In this test, an already prepared reagent is added to a concentrated sputum pellet/isolate to liquefy the specimen and destroy/inactivate any available mycobacteria. The cartridge, attached to the GeneXpert machine, is loaded into the analyzer, which subsequently detects the presence of TB DNA and any mutations that confer rifampin resistance. Internal controls are present within each cartridge to assess the presence of inhibitors that may cause false negative results [10,13]. In this study, for Xpert MTB/ RIF assay, the sample was mixed with sample reagent buffer in 1: 2 (sample: sample reagent buffer) volume ratio and further processed as earlier described and results obtained [13].

**Culture:** for culture, samples were analysed using 3% N-Acetyl-L-Cysteine-Sodium Hydroxide (NALC-NaOH) decontamination method as earlier described [14]. Briefly, the same volume of NALC-NaOH-sodium citrate solution and the sample were mixed, vortexed and left at 25°C for 15 min to enable the NaOH to decontaminate the content of the sputum. Thereafter, 6.8 pH phosphate buffer solution (PBS) was added to the solution, to neutralize and stop the activity of NaOH. The solution was then centrifuged at 3,000 x g for 15 min and 4-8 °C to concentrate the freed bacilli. Finally, a smear was made from the sediment on an already prepared paired egg-based Lowenstein Jensen medium (one containing glycerol and the other pyruvate).

**Data analysis:** data obtained from each participant, using the form, and the laboratory assays were entered into Microsoft Excel®, coded and cleaned up, after which it was exported to STATA version 12 statistical software. Socio-demographic characteristics were presented in frequencies and proportion. The association between the socio-demographic variables and outcome measure (culture and drug susceptibility condition assay) were analysed using the chi-square statistic (bivariate analysis). Multivariate adjusted logistic regression was carried out using variables that were statistically significant at 30% at the bivariate analysis, to accommodate more variables into the logistic regression, in order to identify important risk factors for DR-TB among the study participants. All tests were two-tailed and statistical significance was set at α0.05.

**Ethical clearance:** this was obtained from the University of Ibadan/University College Hospital Institutional Review Board with reference number UI/EC/17/0121.

## Results

**Socio-demographic characteristics of study participants:** overall, 2169 individuals had epidemiologically relevant data available for analysis. More than half (52.14%) of the study participants were female; most (38.36%) were within the age range of 20-39 years. Majority of the participants fell within the category of previously treated (61.96%), while the least was within the ‘other’ category (17.01%). One hundred and sixty-four (7.56%) of the participants were HIV positive, 1392 (64.18%) were DS-TB and 47 (2.17%) of the participants were DR-TB (Table 1).

**Table 1:** socio-demographic variables of study participants in southwestern Nigeria, October 2020 - February 2021

Variables	Frequency (n)	Percentage (%)
**Gender**		
Male	1038	47.86
Female	1131	52.14
**Age group**		
0 - 19	165	7.61
20 - 39	832	38.36
40 - 59	801	36.93
≥ 60	371	17.10
**Case definition**		
New	423	19.50
Relapse	33	1.52
Previously treated	1344	61.96
Others*	369	17.01
**HIV status**		
Positive	164	7.56
Negative	2005	92.44
**Drug susceptibility condition**		
Negative	730	33.66
DS-TB	1392	64.18
DR-TB	47	2.17

Others*: those with unknown outcomes after being previously treated for TB; DS: drug-susceptible; DR: drug-resistant

**Figure 2:**
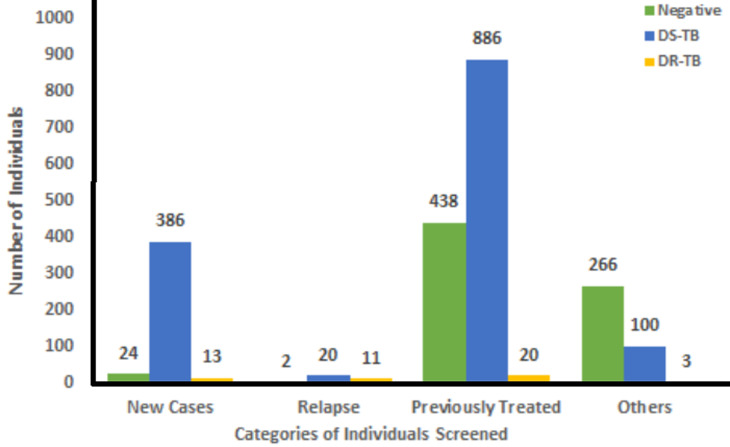
total number (%) of drug-susceptible TB, drug-resistant TB and negatives among different categories of individuals screened

**Prevalence and risk factors of TB among study participants:** in all, the prevalence of TB among presumptive cases was 66.34% by Xpert MTB/RIF assay, 39.42% by culture as well as 4.29% by X-ray (Table 2). However, 105 (4.84%) samples processed by culture were contaminated. Overall, out of the 1439 (66.34%) samples identified as mycobacteria by GeneXpert, 47 (3.27%) were found to be DR-TB and 1392 (96.73%) DS-TB. Also, 3.1% (20/423) and 1.5% (20/1344) were DR-TB among the new cases and previously treated individuals, respectively (Figure 2).

**Table 2:** results of diagnostic tools among screened presumptive TB patients, October 2020 - February 2021

Diagnostic tool	Positive result n (%)	Negative result n (%)	Not done n (%)	Total n (%)
Xpert MTB/RIF assay	1439 (66.34)	730 (33.66)	NA	2169 (100)
Culture result	855 (39.42)	1314 (60.58)	NA	2169 (100)
X-ray	93 (4.29)	8 (0.37)	2068 (95.34)	2169 (100)

NA: not applicable

Based on culture, an association was observed between infection with *Mycobacteria spp*. and gender, age, case definition, HIV status as well as location (Table 3). Female participants were less likely (OR=0.76; 95%CI: 0.64-0.91) to be infected compared to males. Individuals above 60 years (OR=0.69; 95%CI: 0.47-0.95) showed less likelihood of being infected, compared to those below 20 years (Table 3). Those categorized as previously treated (OR=2.38; 95%CI: 1.92-3.03) and others (OR=3.85; 95%CI: 2.78-5.26) were more likely to have DR-TB infection compared to those classified as new cases. Further, participants who were HIV positive are more likely (OR=1.85;95% CI: 1.29-2.65) to be infected compared to HIV-negative individuals (Table 3).

**Table 3:** association between variables and outcome measure (culture and GeneXpert) among participants screened, October 2020 - February 2021

Variables	Culture				GeneXpert			
	Positive n (%) (n=275)	Negative n (%) (n=452)	OR (95%CI)	P-value	Positive n (%) (n=298)	Negative n (%) (n=429)	OR (95%CI)	P-value
**Gender**								
Male	444 (51.93)	594 (45.21)	1		738 (51.29)	300 (41.10)	1	
Female	411 (48.07)	720 (54.79)	0.76 (0.64 - 0.91)	0.00*	701 (48.71)	430 (58.90)	0.66 (0.55 - 0.79)	0.00*
**Age group**								
0 - 19	67 (7.84)	98 (7.46)	1		106 (7.37)	59 (8.08)	1	
20 - 39	352 (41.17)	480 (36.53)	1.07 (0.76 - 1.51)	0.75	589 (40.93)	243 (33.29)	1.35 (0.95 - 1.92)	0.11
40 - 59	316 (36.96)	485 (36.91)	0.95 (0.67 - 1.34)	0.85	519 (36.07)	282 (38.63)	1.02 (0.72 - 1.45)	0.96
≥ 60	120 (14.04)	251 (19.10)	0.69 (0.47 - 0.95)	0.04*	225 (15.64)	146 (20.00)	0.85 (0.58 - 1.26)	0.49
**Case definition**								
New	245 (28.65)	178 (13.55)	1		399 (27.73)	24 (3.29)	1	
Relapse	23 (2.69)	10 (0.76)	1.67 (0.78 - 3.59)	0.26	31 (2.15)	2 (0.27)	0.93 (0.21 - 4.13)	0.77
Previously treated	489 (57.19)	855 (65.07)	2.38 (1.92 - 3.03)	0.00*	906 (62.96)	438 (60.00)	8.33 (5.26 - 12.50)	0.00*
Others**	98 (11.46)	271 (20.62)	3.85 (2.78 - 5.26)	0.00*	103 (27.91)	266 (36.44)	46.67 (27.03 - 71.43)	0.00*
HIV status								
Positive	44 (5.15)	120 (9.13)	1.85 (1.29 - 2.65)	0.00*	62 (4.31)	102 (13.97)	3.61 (2.59 - 5.02)	0.00*
Negative	811 (94.85)	1194 (90.87)	1		1377 (95.69)	628 (86.03)	1	

Others**: those with unknown outcomes after being previously treated for TB; *significant association at p≤0.05

Using GeneXpert, an association was observed between TB infection and gender, case definition, HIV status as well as location. Female participants are less likely (OR=0.66; 95%CI: 0.55-0.79) to be infected with TB compared to males. Individuals categorized as previously treated (OR=8.33; 95%CI: 5.26- 12.50) and others (OR=46.67; 95%CI: 27.03-71.43) are more likely to be infected with DR-TB (Table 3).

**Associated risk factor for TB infection among study participants:** the multivariate-adjusted logistic regression analysis identified case definition (participant that falls within the category of new cases, relapse cases, previously treated cases, and others {those with unknown outcome after been previously treated for TB}) and HIV status based on culture as important associated risk factors for the occurrence of DR-TB infection among the study participants (Table 4). Individuals who were identified as previously treated cases (AOR=2.27.49 95%CI: 1.82-2.86; P=0.00), and those categorized as other cases (AOR=3.57; 95%CI: 2.63-4.76; P=0.00) were about two and four times more likely to be infected with DR-TB infection compared to the newly diagnosed cases. Participants who were infected with HIV are 1.66 times more likely (AOR=1.66 95%CI: 1.55 - 2.39; P=0.01) to be infected with DR-TB compared to HIV-negative individuals (Table 4).

**Table 4:** associated risk factors for the TB infection in study participants based on culture as diagnostic techniques, October 2020 - February 2021

Variable	Adjusted odd ratio	Std error	Z-value	P-value	95% CI
**Gender**					
**Male**	**Reference group**				
Female	0.87	0.08	-1.43	0.15	0.73 - 1.05
**Age group**					
**0 - 19**	**Reference group**				
20 - 39	1.02	0.18	0.13	0.89	0.72 -1.45
40 - 59	0.94	0.17	-0.32	0.74	0.67 - 1.34
≥ 60	0.74	0.15	-1.51	0.13	0.50 - 1.09
**Case definition**					
**New**	**Reference group**				
Relapse	1.58	0.62	1.17	0.24	0.73 - 3.41
Previously treated	2.27	0.05	-7.13	0.00*	1.82 - 2.86
Others**	3.57	0.04	-8.16	0.00*	2.63 - 4.76
**HIV status**					
Positive	1.66	0.31	2.73	0.01*	1.15 - 2.39
Negative	Reference group				

Others**: those with unknown outcomes after being previously treated for TB; *significant association at p≤0.05

Based on GeneXpert, the multivariate-adjusted logistic regression identified case definition, and HIV status as important associated risk factors for the occurrence of TB infection among the study participants (Table 5). Participants categorized as previously treated (AOR=7.69; 95%CI: 4.76-11.11) and others (AOR=41.64; 95%CI: 25.64-66.67) were 8 and 41 times more likely to have DR-TB compared to those identified as new cases. Further, individuals who were HIV positive were almost four times more likely (AOR=3.69 95%CI: 2.57 - 5.29; P=0.00) to be infected with DR-TB compared to HIV-negative individuals (Table 5).

**Table 5:** associated risk factors for TB infection in study participants based on GeneXpert as diagnostic techniques, October 2020 - February 2021

Variable	Adjusted odd ratio	Std error	Z-value	P-value	95% CI
**Gender**					
**Male**	**Reference group**				
Female	0.85	0.09	-1.50	0.13	0.69 - 1.04
**Age group**					
**0 - 19**	**Reference group**				
20 - 39	1.27	0.26	1.16	0.24	0.85 - 1.89
40 - 59	0.99	0.19	-0.06	0.95	0.66 - 1.47
≥ 60	0.90	0.19	-20.48	0.63	0.58 - 1.38
**Case definition**					
**New**	**Reference group**				
Relapse	1.23	0.78	2.09	0.78	0.18 - 3.61
Previously treated	7.69	0.00	-18.95	0.00*	4.76 - 11.11
Others	41.64	0.00	-10.50	0.00*	25.64 - 66.67
**HIV status**					
Positive	3.69	0.68	7.07	0.00*	2.57 - 5.29
Negative	Reference group				

## Discussion

This study recorded a prevalence of 2.17% for rifampicin-resistant TB among presumptive TB patients attending various DOTS centers in the study areas. The findings of this study, though a preliminary part of an ongoing large-scale epidemiological and mycobacteria genomic study underscore the importance and usefulness of GeneXpert in the diagnostic regimen of DR-TB. According to the WHO 2020 TB annual report, the prevalence of TB in the African region is still high, with millions of cases remaining undiagnosed. This situation is exacerbated by the emergence of MDR-TB [9]. Notably, the diagnosis of TB, DR-TB, and MDR-TB in Nigeria, like in most LMICs, still largely depends on highly limited smear microscopy. Consequently, there is an increased prevalence of smear-negative pulmonary TB patients who are clinically infected, which persists in the population undiagnosed or experiencing delayed diagnosis and treatment. Therefore, identifying the true burden of TB, including DR-TB, will go a long way in contributing to the development of informed decisions and/or policies for effective and efficient control measures geared towards the eradication of the disease globally.

Importantly, the prevalence of RR-TB recorded in this study is less than the range of 3.2-5.4% that WHO predicted for Nigeria and 3.3% reported by Otokunefor *et al*. (2018) [15], in Port Harcourt, south-southern Nigeria. Also, it is less than 7.3% reported by Ukwamedua *et al*. (2019) [16], in South-South Nigeria; 10% in Adamawa, North-East Nigeria by Omisore *et al*. (2018) [17]; 7.4% reported in Southwestern Nigeria by Kuyinu *et al*. 2018 [18], and 10.8% in Southeastern Nigeria by Ahiarakwem *et al*. (2022) [19]; 19.0% reported in Northern Nigeria [20]; 14.7% reported in South-south Nigeria [21], 21.3% in Southeastern Nigeria [22], and 32.0% reported in a systematic review and meta-analysis carried out in Nigeria [23]. In a recent retrospective study conducted by Oladimeji *et al*. (2022) [24] across the country, RR prevalence of 6.47% was recorded in North-east; 14.12% in North-central; 31.18% in North-west; 10.59% in South-south; 3.53% in South-East, and 34.12% in south-west; from the available database of DR-TB patients managed in specialized DR-TB treatment facilities in the six geopolitical zones of Nigeria [25]. The difference in the variously reported prevalence vis-à-vis that of the current studies could be attributed to various factors and issues that border around systemic and systematic variations associated with individual studies, such as differences in methodology, sample size, study population and settings, time of the study execution, accessibility and availability of diagnostic facilities especially in more rural terrains, socio-political issues that militate against patients' initiation and continuation of the treatment regimen, and many others [13,23]. Our findings revealed a high prevalence of TB and a moderately high prevalence of DR-TB among the study participants. The high prevalence recorded in this study underscores the endemicity of disease in the study areas. It should be noted that resistance to rifampicin has grave implications for TB management and control policies, since 80% of RR-TB is also MDR/RR-TB; and RIF resistance has been reported to predict and/or serve as a precursor to MDR-TB infection [25,26]. Further, the discordance observed between the GeneXpert and culture results may not be unconnected with the higher sensitivity of GeneXpert, as it could detect dead remnant DNA from retreatment patients. Again, the harsh process of decontamination and contamination encountered during culture growth are other important factors. These assertions have been previously documented [14]. Again, the quality of the sputum samples processed is also important since poor storage prior to transportation and delivery at the laboratory may also have compromised the sputum samples used for culture.

Our findings further identified case definition and HIV status as important risk factors for TB infection among the population screened. Again, individuals previously treated and others (those with unknown outcomes after being previously treated for TB) were more likely to be infected or have persistent infection. The obvious reason for this is not far-fetched. Notably, individuals with infections who fail to return for treatment after the initial visit will continue to harbour the disease since they have not been exposed to the treatment regimen for TB. Again, previously treated, but not completely cured individuals will still have the infections. Apart from this, these groups of people are prone to developing DR and MDR-TB [27,28]. Individuals with HIV are more likely to be infected compared to non-infected participants.

Immunocompromising conditions such as poor nutritional status and co-morbidities like HIV have been associated with high risk for TB infection. A known major risk factor for increased and persistent incidence of TB, especially in LMIC is HIV infection [17]. There have been reports of dynamic interaction between TB and HIV infection; people living with HIV (PLHIV) have increased susceptibility to TB infection, and HIV has been linked to treatment failure of TB-control programmes in attaining targets, most especially in high-burdened regions [29]. HIV co-infection has been reported to complicate the situation of TB/DR-TB/MDR/XDR-TB and is considered a major threat with challenging case management [29]. In line with the aforementioned, it was observed that most of the HIV-infected individuals were those with previously treated TB.

The findings from the culture and GeneXpert results indicate that females have a lower likelihood of being infected with DR-TB. This observation holds true even when potential confounding factors were considered at the adjusted model, suggesting that gender plays a significant role in determining the risk of DR-TB infection. Notably, this pattern is similar among individuals who have previously undergone TB treatment and among those who were HIV positive. Among individuals who have been previously treated for TB, as well as among HIV-positive patients, the likelihood of having DR-TB infection was consistently higher. The culture and GeneXpert results consistently demonstrated that these two factors were associated with an increased risk of DR-TB infection.

Taken together, the findings from this study provide compelling evidence that gender, previous TB treatment, and HIV status are significant factors that influence the likelihood of acquiring DR-TB infection. These results highlight the importance of considering these factors in the prevention, diagnosis, and treatment strategies for DR-TB.

**Limitations:** despite the findings observed, this study had some limitations. First, the study was carried out among presumptive patients attending DOTs facilities, thus it might not be a true representation of the situation of TB infection in the general population. However, the current data provides important information on DR-TB in the study area. Further, only RR was investigated (using GeneXpert), thus further phenotypic and genotypic analyses would have provided better insight into the DR pattern for the MDR/XDR-TB scenario. However, other relevant analyses are ongoing that will provide further epidemiological and genomic insights.

Overall, the findings from this study provide compelling evidence that gender, previous TB treatment, and HIV status are significant factors that influence the likelihood of acquiring DR-TB infection. These results highlight the importance of considering these factors in the prevention, diagnosis, and treatment strategies for DR-TB. Generally, the prevalence of TB and DR-TB was found to be high among the studied population in Southwestern Nigeria. This underscores the need for more intense testing, strengthening, and establishment of more viable control programs with strict monitoring of patients and an emphasis on compliance and completion of treatment. As earlier stated, this is an initial part of an ongoing more comprehensive study, thus further analyses and epidemiological evaluations would be required to shed more light on the transmission patterns and other epidemiological variables associated with drug-resistant TB in the study locations. This will serve as a model for a more in-depth study for the whole country, considering the endemicity of the disease in Nigeria.

## Conclusion

The findings from the culture and GeneXpert results indicate that females have a lower likelihood of being infected with DR-TB. This observation holds true even when potential confounding factors were considered in the adjusted model, suggesting that gender plays a significant role in determining the risk of DR-TB infection. Notably, this pattern is similar among individuals who have previously undergone TB treatment and those who are HIV positive. Among individuals who have been previously treated for TB, as well as among HIV-positive patients, the likelihood of having DR-TB infection was consistently higher. The culture and GeneXpert results consistently demonstrated that these two factors are associated with an increased risk of DR-TB infection.

### What is known about this topic

- Tuberculosis is a global health challenge, with far-reaching effects in low and middle-income countries;
- The emergence of DR-TB poses a major threat to the global effort to curtail TB;
- Nigeria is among eight countries with the highest prevalence of TB globally, and an unknown burden of MDR-TB.

### What this study adds

- The study reported a moderate level prevalence of DR-TB (Rifampicin resistance) among the study population;
- Gender, previous treatment, and HIV status are the important risk factors for DR-TB identified in this study;
- The combination of culture and GeneXpert was identified as the optimum diagnostic test for TB in LMIC.
